# Binding of perilipin 3 to membranes containing diacylglycerol is mediated by conserved residues within its PAT domain

**DOI:** 10.1016/j.jbc.2023.105384

**Published:** 2023-10-28

**Authors:** Jiri Stribny, Roger Schneiter

**Affiliations:** Department of Biology, University of Fribourg, Fribourg, Switzerland

**Keywords:** perilipin, diacylglycerol, lipid binding, lipid droplets, microscale thermophoresis, liposomes, artificial lipid droplets, short-chain lipids, PAT domain, apolipoprotein

## Abstract

Perilipins (PLINs) constitute an evolutionarily conserved family of proteins that specifically associate with the surface of lipid droplets (LDs). These proteins function in LD biogenesis and lipolysis and help to stabilize the surface of LDs. PLINs are typically composed of three different protein domains. They share an N-terminal PAT domain of unknown structure and function, a central region containing 11-mer repeats that form amphipathic helices, and a C-terminal domain that adopts a 4-helix bundle structure. How exactly these three distinct domains contribute to PLIN function remains to be determined. Here, we show that the N-terminal PAT domain of PLIN3 binds diacylglycerol (DAG), the precursor to triacylglycerol, a major storage lipid of LDs. PLIN3 and its PAT domain alone bind liposomes with micromolar affinity and PLIN3 binds artificial LDs containing low concentrations of DAG with nanomolar affinity. The PAT domain of PLIN3 is predicted to adopt an amphipathic triangular shaped structure. *In silico* ligand docking indicates that DAG binds to one of the highly curved regions within this domain. A conserved aspartic acid residue in the PAT domain, E86, is predicted to interact with DAG, and we found that its substitution abrogates high affinity binding of DAG as well as DAG-stimulated association with liposome and artificial LDs. These results indicate that the PAT domain of PLINs harbor specific lipid-binding properties that are important for targeting these proteins to the surface of LDs and to ER membrane domains enriched in DAG to promote LD formation.

Most cells store metabolic energy in the form of neutral lipids within dedicated intracellular lipid droplets (LDs). LDs are composed of a core of neutral lipids, particularly triacylglycerol (TAG) and steryl esters (STE), which is shielded from the aqueous environment by a monolayer coat of phospholipid. A set of proteins, which specifically localize to the LD surface, serve to functionalize the compartment during lipogenesis and lipolysis. Many of these LD-localized proteins function in neutral lipid metabolism, serving as acyltransferases or lipases, and are thus implicated in energy and membrane homeostasis (1, 2). In addition to these metabolic enzymes, the LD surface is covered by perilipins (PLINs), a family of soluble proteins that specifically coat the surface of LDs. These abundant proteins are generally thought to stabilize LDs, promote LD formation, and to regulate lipolysis (3, 4, 5). This regulation of neutral lipid metabolism is critical for maintaining energy balance, as its disruption can lead to metabolic disorders, including obesity and diabetes (6).

Mammals express five different PLIN family members (PLIN1-PLIN5), which are either expressed ubiquitously (PLIN2/ADRP, PLIN3/TIP47) or in a tissue-specific manner (PLIN1, PLIN4/S3-12, PLIN5/OXPAT), particularly in adipocyte and steroidogenic tissue (4, 7). Several studies have sought to unravel how exactly PLINs are targeted to the surface of LDs. However, the exact mechanism of this targeting and the specificity of the interaction with the LD surface remains to be fully elucidated (8, 9, 10).

Most of the PLIN proteins are characterized by the presence of three conserved domains. Except for PLIN4, all PLINs have an N-terminal PAT (PLIN1, PLIN2/ADRP, PLIN3/TIP47) domain of unknown structure or function (11). The PAT domain is then followed by a domain composed of repeats of 11 amino acids (11-mer), which form amphipathic helices (AHs). These AHs have been proposed to recognize lipid-packaging defects and to target PLINs and other proteins-bearing AHs, including apolipoproteins and α-synuclein, to the LD surface (9, 12, 13, 14, 15).

The C-terminal domain of PLIN2, 3, and 5 harbors a bundle of four long helices (4-helix bundle), similar to those present in apolipoprotein E and the insect hemolymph apolipophorin III (16, 17). This 4-helix bundle domain has been implicated in forming lipidic discs *in vitro* (18). The precise role and contribution of these three individual domains, PAT, 11-mer repeats, and the 4-helix bundle, to the overall function of PLINs, however, is poorly understood.

Here, we characterize the lipid-binding properties of PLIN3. We show that the N-terminal PAT domain of PLIN3 binds diacylglycerol (DAG) and promotes association of PLIN3 with liposomes containing low concentrations of DAG. Using structural predictions of the PAT domain by AlphaFold2 combined with *in silico* lipid docking analysis, we identify a potential DAG-binding site within one of the highly curved regions of the triangular-shaped PAT domain. Mutagenesis of key residues predicted to interact with DAG validate their functional importance in DAG binding and in promoting association of PLIN3 with DAG-containing liposomes and artificial LDs (ALDs). The observation that the PAT domain of PLIN3 binds DAG, the immediate precursor to TAG, provides for a possible molecular mechanism of this protein in promoting LD formation at the membrane of the endoplasmic reticulum (ER) and in localizing to and stabilizing the surface of mature LDs.

## Results

### PLIN3 binds liposomes containing low concentrations of DAG

**Figure 1:**
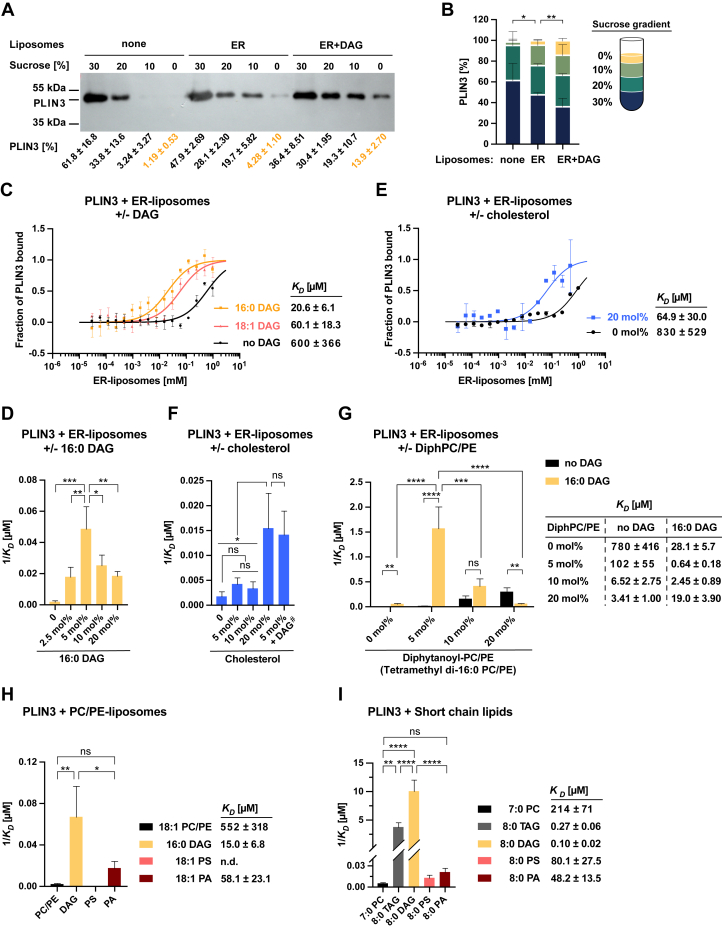
**PLIN3 harbors enhanced binding affinity towards ER-liposomes containing DAG.***A*, PLIN3 recruitment to ER-liposomes composed of DOPC (53 mol%), DOPE (23 mol%), DOPS (8 mol%), DOPA (5 mol%), and SoyPI (11 mol%) and to ER-liposomes supplemented with 16:0 DAG (5 mol%), assayed by flotation on a sucrose gradient. Liposome-bound PLIN3 was analyzed by Western blotting using an anti-His antibody, and signals of individual fractions were quantified (mean ± S.D. of three independent experiments). *B*, graph of PLIN3 distribution in sucrose fractions. The statistical significance of liposome-bound PLIN3 recovered from the *top* layer of the sucrose gradient (*yellow boxes*) is indicated. ∗*p* < 0.05; ∗∗*p* < 0.01 (two-tailed unpaired *t* test). *C*, PLIN3 binding to ER-liposomes supplemented either with 16:0 DAG or 18:1 DAG (5 mol%) measured by MST. The fraction of liposome-bound protein is plotted against the liposome concentration and the dissociation constant (*K*_*D*_) is indicated. *D*, dose-dependence of PLIN3 binding to ER-liposomes containing increasing concentrations of 16:0 DAG (2.5–20 mol%). *E* and *F*, PLIN3 binding to ER-liposomes supplemented with different concentrations of cholesterol or with cholesterol and DAG together (#; both at 5 mol%). *G*, PLIN3 binding to ER-liposomes containing different concentrations of both diphytanoyl-PC and diphytanoyl-PE (combined concentration of 5, 10, 20 mol%), either lacking or containing DAG (5 mol%). *H*, PLIN3 binding to PC/PE liposomes (DOPC/DOPE; 80/20 mol%) spiked with either DAG (16:0 DAG; 5 mol%), PS (DOPS; 10 mol%), or PA (DOPA; 10 mol%). *I*, PLIN3 binding to the indicated short chain lipids. Dissociation constants (*K*_*D*_) are plotted as reciprocal values (*panels D*, *F*–*I*). All values represent mean ± S.D. of three independent measurements. ∗*p* < 0.05; ∗∗*p* < 0.01; ∗∗∗*p* < 0.001; ∗∗∗∗*p* < 0.0001; ns, not significant (one-way ANOVA with Tukey's post hoc test); n.d., not detected. DAG, diacylglycerol; ER, endoplasmic reticulum; MST, microscale thermophoresis; PA, phosphatidic acid; PC, phosphatidylcholine; PE, phosphatidylethanolamine; PLIN, perilipin.

DAG has previously been shown to be required for LD formation and to accumulate at ER subdomains engaged in LD formation (19, 20, 21, 22, 23). To examine the interaction between soluble PLIN3 and membranes containing DAG, we first performed liposome-binding assays. Therefore, purified His-tagged human PLIN3 was incubated with liposomes prepared from a phospholipid mixture that mimics the composition of the ER membrane (DOPC/DOPE/DOPS/DOPA/SoyPI, 53/23/8/5/11 mol%; hereafter termed ER-liposomes) lacking or containing low concentrations of DAG (16:0 DAG; 5 mol%). Liposomes were then floated on a sucrose gradient, and PLIN3 binding was monitored by Western blotting. Compared to ER-liposomes lacking DAG (4.28% ± 1.10 of PLIN3), a significantly higher fraction of PLIN3 was recovered from ER-liposomes containing DAG (13.9% ± 2.70), suggesting that the presence of low concentrations of DAG in these membranes stimulates liposome binding of PLIN3 (Fig. 1, *A* and *B*). Precise *p*-values of all statistical analyses are given in Tables S1 and S2.

To corroborate these findings, we analyzed binding of PLIN3 to liposomes using microscale thermophoresis (MST), an immobilization-free technology for measuring biomolecular interactions based on the diffusion of a fluorescently labeled protein along a microscopic temperature gradient. In this assay, the free protein diffuses faster than the one which has bound to a ligand. Ligands can range from small ions to low molecular weight compounds, liposomes, or even viruses (24, 25, 26, 27, 28). Variations in fluorescence intensity are then plotted against the ligand concentrations to determine a binding affinity. PLIN3 displayed a 29-fold lower dissociation constant (*K*_*D*_ of 20.6 μM) to ER-liposomes containing low concentrations of DAG (16:0 DAG; 5 mol%) than ER-liposomes lacking DAG (*K*_*D*_ of 600 μM). Enhanced binding of PLIN3 to DAG-containing liposomes was also observed when liposomes were spiked with a DAG species harboring two unsaturated acyl chains (18:1 DAG; 5 mol%; *K*_*D*_ of 60.1 μM), indicating that the precise acyl chain composition of DAG is not a main determinant for the increased binding affinity (Fig. 1*C*). To determine the optimal DAG concentrations for PLIN3 binding, we varied the DAG concentrations in the ER-liposomes between 0 to 20 mol%. PLIN3 displayed lowest *K*_*D*_ to ER-liposomes containing 5 mol% DAG, as higher DAG concentrations did not result in a further enhancement of the binding affinity (Fig. 1*D*).

The observed low concentration–induced stimulation of PLIN3 binding to ER-liposomes was specific for the presence of DAG in the membrane as incorporation of a similarly low concentration of cholesterol into ER-liposomes did not result in a comparable enhancement of PLIN3 binding (Fig. 1*F*). Higher concentrations of cholesterol, however, also promoted binding of PLIN3 (20 mol% cholesterol, *K*_*D*_ of 64.9 μM; Fig. 1*E*). Spiking the ER-liposomes with both DAG and cholesterol, on the other hand, did not result in a synergistic effect of the two lipids in promoting the binding of PLIN3 (Fig. 1*F*). The cholesterol concentration in the ER membrane of mammalian cells has been estimated at 5 mol% whereas that of the plasma membrane is much higher (20 mol%) (29). Given that PLIN3 is localized to the ER membrane and to LDs and that a cholesterol concentration as is typically found in the ER membrane did not enhance binding of PLIN3, we did not further investigate a possible role of cholesterol in promoting membrane association of PLIN3, even though PLIN2 has been shown to bind fluorescent NBD-cholesterol with high affinity through its 4-helix bundle domain (30, 31).

Diphytanoyl lipids, that is, lipids containing methyl-branched acyl chains, have previously been shown to increase binding of PLIN4 to liposomes (13). PLIN4 is an unusual PLIN because it contains an extremely long AH but lacks both the PAT domain and the 4-helix bundle. To test whether the presence of diphytanoyl lipids would also enhance binding of PLIN3 to liposomes, ER-liposomes were spiked with both diphytanoyl-phosphatidylcholine (PC) and diphytanoyl-phosphatidylethanolamine (PE), at combined concentrations of 5, 10, or 20 mol%. This addition of diphytanoyl-PC/PE resulted in increased binding of PLIN3 to ER-liposomes, but at low concentrations, did not abolish DAG-stimulated membrane binding of the protein (Fig. 1*G*). Taken together, these results indicate that PLIN3 displays increased affinity towards bilayer membranes containing low concentrations of DAG. DAG serves as immediate metabolic precursor to the formation of TAG, a major storage lipid of LDs. Thus, DAG binding by PLIN3 in the ER membrane may serve to promote LD formation at specialized ER domains (21, 32).

### Defining the lipid specificity for increased membrane binding of PLIN3

To better define the lipid-selectivity for the observed increase of membrane binding by PLIN3, we first reduced the phospholipid complexity of the liposomes, and instead of ER-liposomes, we prepared liposomes composed of the major two phospholipids present in the ER membrane, PC and PE. These DOPC/DOPE (80/20 mol%) liposomes were then spiked with either DAG (16:0 DAG; 5 mol%), phosphatidylserine (DOPS), or phosphatidic acid (PA) (DOPA; at 10 mol% each), and liposome binding by PLIN3 was determined by MST. Again, in the presence of DAG, PLIN3 binding was strongly increased (36-fold, *K*_*D*_ 15 μM *versus* 552 μM) (Fig. 1*H*). While the addition of DOPS to liposomes did not affect PLIN3 binding, the presence of DOPA enhanced binding by (9-fold, *K*_*D*_ 58.1 μM *versus* 552 μM). These results indicate that low concentrations of DAG increased membrane binding of PLIN3 not only in complex ER-liposomes, which contain DOPA (5 mol%), but that a similar stimulation of membrane binding was also observed with simpler liposomes composed of DOPC and DOPE only.

To examine whether PLIN3 would directly bind DAG or whether the enhanced binding to DAG-containing liposomes is due to DAG-induced alterations in lipid packaging, we tested binding of more soluble, saturated short-chain 7:0- and 8:0-lipids. PLIN3 bound both 8:0 DAG (*K*_*D*_ of 0.10 μM) and 8:0 TAG (*K*_*D*_ of 0.27 μM) with submicromolar affinity (Fig. 1*I*). The short chain phospholipids, on the other hand, showed much higher (>480-fold) dissociation constants, suggesting that the presence of a negatively charged phosphate group is unfavorable for binding and that PLIN3 strongly prefers to bind neutral lipids such as DAG or TAG over phospholipids. When combined with the results from the liposome-binding assays, these data suggests that PLIN3 harbors affinity for noncharged glycerolipids, either when provided as short-chain lipid ligands or, as is the case of DAG, when the neutral lipid is embedded within a lipid bilayer.

### Binding to DAG-containing liposomes is mediated by the PAT domain of PLIN3

**Figure 2:**
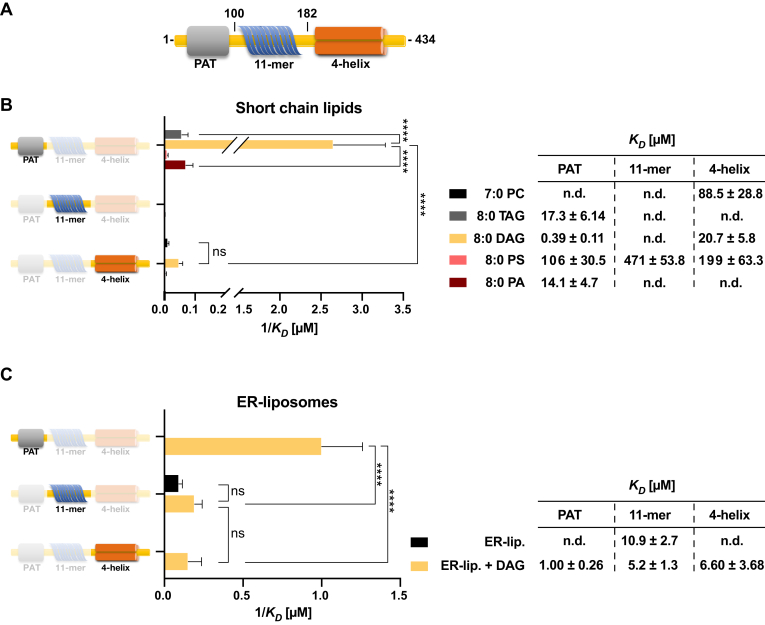
**The PAT domain of PLIN3 binds short chain DAG and has high binding affinity towards liposomes containing DAG.***A*, domain organization of PLIN3. Amino acid positions used to express single domain proteins are indicated. *B* and *C*, lipid-binding specificity of individual domains of PLIN3 were assessed with short chain lipids (*B*) or ER-liposomes lacking or containing 16:0 DAG (5 mol%; composition as in Fig. 1) (*C*). Dissociation constants (*K*_*D*_) are plotted as reciprocal values (mean ± S.D. of three independent measurements). ∗∗∗∗*p* < 0.0001; ns, not significant (two-way ANOVA with Tukey's post hoc test); n.d., not detected. DAG, diacylglycerol; ER, endoplasmic reticulum; PLIN, perilipin.

To gain further insight into the binding of PLIN3 to DAG-containing membranes, we examined the contribution of each individual domain of PLIN3 towards lipid binding, that is, the N-terminal PAT domain, the central 11-mer repeat region, and the C-terminally located 4-helix bundle (Fig. 2*A*). Therefore, we expressed and purified proteins harboring individual domains of PLIN3 and first examined their binding of short-chain lipids. This comparative analysis revealed that the PAT domain of PLIN3 bound 8:0 DAG with the highest affinity (*K*_*D*_ of 0.39 μM; Fig. 2*B*). Consistent with short chain DAG binding to full-length PLIN3, DAG binding by the PAT domain was 36-fold stronger than was binding of 8:0 PA (*K*_*D*_ of 14.1 μM), which was followed by binding of 8:0 TAG (*K*_*D*_ of 17.3 μM). The 11-mer AH repeat region and the 4-helix bundle did not display submicromolar-binding affinities to any of the short-chain lipids tested. Noteworthy, however, the 4-helix bundle bound 8:0 DAG with a *K*_*D*_ of 20.7 μM, suggestive of a possible synergistic action of these two domains towards lipid-binding of PLIN3 (Fig. 2*B*).

To validate the binding specificity of the individual domains of PLIN3 towards short chain lipids, we determined their binding to ER-liposomes lacking or containing low concentrations of DAG (5 mol%). Compared to the 11-mer repeats or the 4-helix bundle, the PAT domain displayed the highest binding affinity (*K*_*D*_ of 1.0 μM). However, even the 11-mer AH repeat and the 4-helix bundle exhibited increased micromolar-binding affinities towards DAG-containing ER-liposomes compared to liposomes lacking DAG, indicating that low concentrations of DAG promote bilayer binding of all three domains of PLIN3 (Fig. 2*C*). Taken together, these results indicate that the N-terminal PAT domain of PLIN3 binds both short chain DAG and harbors a strong binding specificity towards liposomes containing DAG.

### The PAT domain adopts a conserved amphipathic triangular helical topology

**Figure 3:**
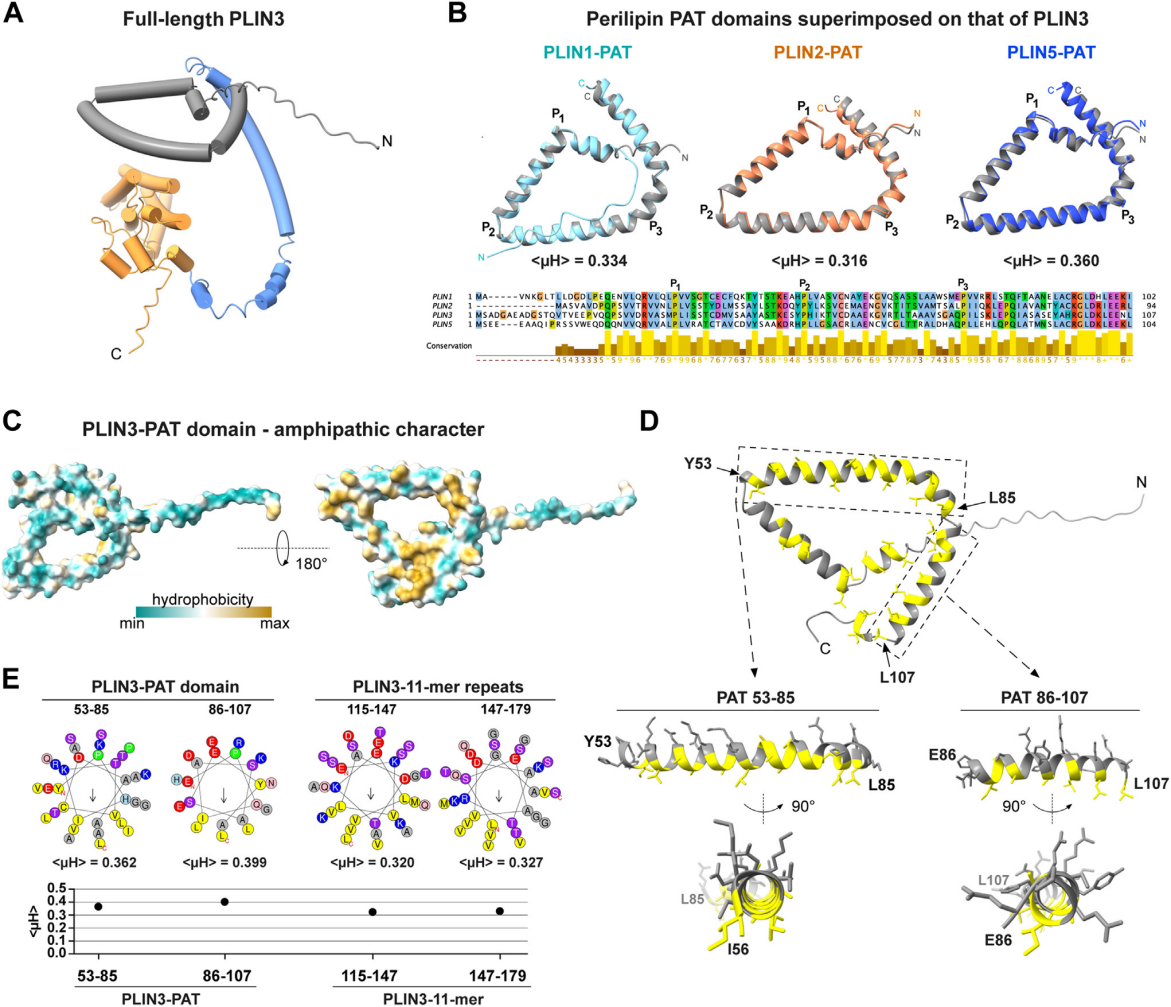
**The PAT domain of PLIN3 adopts a triangular-shaped amphipathic structure.***A*, cartoon of the structure of PLIN3 as predicted by AlphaFold2, depicting a simplified representation of individual domains of PLIN3 with helical structures drawn as cylinders (PAT, *gray*; 11-mer repeats, *blue*; 4-helix bundle, *orange*). *B*, *upper panel*, superimposition of the structure of the PAT domain of PLIN3 (*gray*) with PAT domains of the indicated PLIN family members (PLIN1, 2, 5) illustrating conservation of the predicted triangular topology. Structures were predicted by AlphaFold2 and superimposed on the PAT domain of PLIN3 using the MatchMaker alignment function of Chimera X. Their hydrophobic moments (<μH>) as calculated by HeliQuest are indicated. *Lower panel*, multiple sequence alignment of the PAT domains from the indicated PLIN family proteins. The positions of the three conserved proline residues (P1-P3) are indicated. *C*, model of the PAT domain with surface residues colored according to their hydrophobicity with a view of the structure turned by 180°. *D*, visualization of the amphipathic helices (residues 53–85 and 86–107) of the PAT domain with hydrophobic residues highlighted in *yellow*. *E*, HeliQuest analysis comparing the amphipathic helices of the PAT domain (residues 53–85 and 86–107) with that of the 11-mer repeats (residues 115–147 and 147–179). Hydrophobic moments (<μH>) of segments are indicated and plotted below the respective helical wheels. PLIN, perilipin.

Given the lack of an experimentally determined high-resolution structure of full length PLIN3 or of its PAT domain alone, we analyzed the structure of PLIN3 as predicted by AlphaFold2 (33, 34, 35). The PAT domain is predicted to be composed of a long helical segment that adopts a unique triangular-shaped topology (Fig. 3*A*). This triangular-shaped helical topology appears to be a conserved structure of the PAT domain, as it was also predicted for the PAT domains of PLIN1, PLIN2, and PLIN5, all of which can be superimposed onto the triangular shape of the PAT domain of PLIN3 (Fig. 3*B*). Sequence alignment of these PAT domains revealed that the three proline residues (P1-P3) that impose the kinks into the helical structures and thus define the edges of the triangular shape are conserved (Fig. 3*B*). Examination of the distribution of hydrophobic residues over the surface area of the PAT domain of PLIN3 indicates a spatial separation of hydrophobic and polar residues, suggesting that the PAT domain exhibits an amphipathic character (Fig. 3*C*). More detailed analysis of the distribution of hydrophobic residues within the predicted helices illustrates the amphipathic character of the PAT domain (Fig. 3*D*). HeliQuest analysis further confirmed the amphipathic nature of the PAT domain and yields a hydrophobic moment of the PAT domain (<μH > 0.362–0.399) that is comparable to that of the central 11-mer repeat segment (<μH > 0.32) (Fig. 3*E*) (12, 18, 36). The PAT domains of PLIN1, 2, and 5 also exhibit an amphipathic character (<μH > 0.316–0.360) indicating that not only the triangular topology of the domain is conserved but also its amphipathic character (Fig. 3*B*). These data thus suggest that the PAT domain of PLINs promotes membrane association of the full-length proteins by its amphipathic character as has been observed for other types of exchangeable apolipoproteins containing AHs (37).

### Characterization of the DAG-binding site within the PAT domain of PLIN3

**Figure 4:**
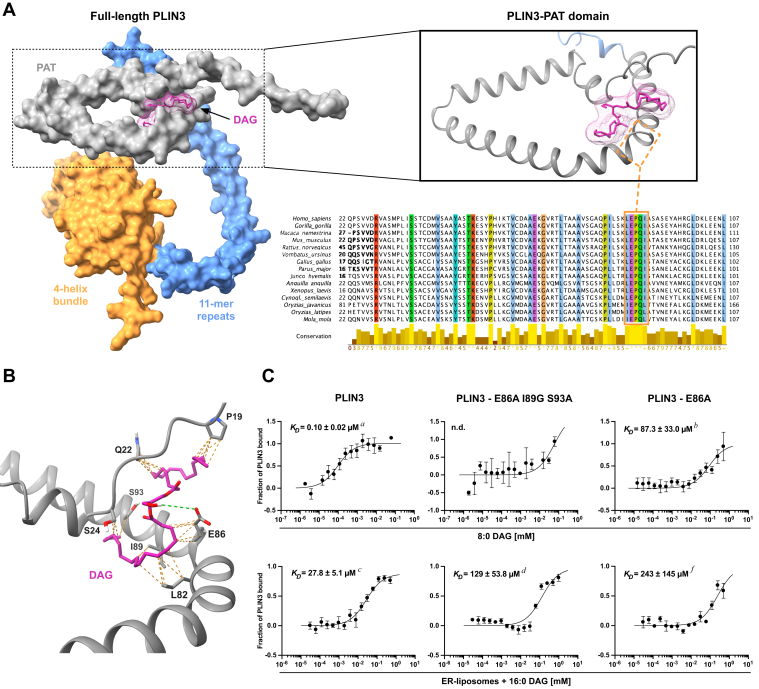
**Highly conserved residues within the PAT domain of PLIN3 are required for binding of DAG.***A*, docking of DAG in the PAT domain of PLIN3. The domain structure of PLIN3 is indicated in colors, PAT (*gray*), 11-mer repeats (*blue*), 4-helix bundle (*orange*), and docking of DAG (*pink*) as predicted by AutoDock Vina is shown. *Upper right-hand* side, detailed view of the predicted DAG binding site at the third kink of the triangular-shaped PAT domain, in proximity of the highly conserved 85-LEPQI-89 motif (*orange box*). *Lower right-hand* side, multiple sequence alignment of PAT domains of PLIN3 orthologs obtained from the OMA database (orthologous matrix; (67)). The sequences of PLIN3 (OMA Group 1153374; Fingerprint STVMSTR) were downloaded, aligned with MAFFT (68), and cleaned with Jalview (69) to highlight conserved residues within the PAT domain. *B*, detailed view of the interactions between DAG (*pink*) and residues within the PAT domain of PLIN3 (van der Waals interactions, *dashed orange lines*; hydrogen bonding, *dashed green line*). *C*, mutations of highly conserved residues within the PAT domain affect binding of DAG. The impact of the indicated mutations on the binding of short chain DAG (8:0 DAG; *upper panels*) or ER-liposomes containing 16:0 DAG (5 mol%; *lower panels*) by PLIN3 were assessed by MST. Dissociation constants (*K*_*D*_) represent mean ± S.D of three independent measurements. (n.d., not detected). *a*, *p* < 0.0001, difference *versus b*; *c*, *p* < 0.05, difference *versus d* and *f*; *d versus f* not significant difference (two-tailed unpaired *t* test). DAG, diacylglycerol; ER, endoplasmic reticulum; MST, microscale thermophoresis; PLIN, perilipin.

To identify a possible DAG-binding site in the PAT domain of PLIN3, we performed *in silico* docking simulations (38, 39). This analysis yielded an energetically favorable docking of DAG within the PAT domain of PLIN3 at the third kink of its triangular-shaped structure (Fig. 4*A*). Interestingly, this third kink contains a highly conserved sequence motif (L/I)EPQ(L/I) as revealed by alignment of PLIN3 orthologs (Fig. 4*A*). Closer analysis of this region identified a set of amino acids that appear to stabilize the interaction with DAG through van der Waals interactions and identified a hydrogen bridge between a glutamic acid residue at position 86 of PLIN3 and the primary hydroxyl of DAG (Fig. 4*B*). Five residues Q22, S24, E86, I89, and S93 appear to be particularly important to stabilize the interaction of PLIN3 with DAG (Fig. 4*B*). Q22 and S24 are located on the short N-terminal tail of PLIN3, which crosses the long-curved helix of the PAT domain from above, at close to rectangular angle. E86 and I89 form part of the highly conserved (L/I)EPQ(L/I) motif.

To validate this predicted mode of DAG binding by the PAT domain, we generated mutant versions of PLIN3 and assessed their impact on DAG binding by MST. First, we tested whether the simultaneous substitution of the five residues, Q22, S24, E86A, I89G, and S93A, would impact either binding of short chain DAG or that of liposomes containing low concentrations of DAG. The quintuple mutant version failed to bind 8:0 DAG or liposomes, indicating that these residues are indeed important for lipid binding (Fig. S1). To assess the impact of these residues in more detail, we first tested mutations in the two serine residues S24A and S93A. This serine double mutant showed a 20.9-fold decreased affinity towards 8:0 DAG (*K*_*D*_ of 2.09 μM *versus* 0.1 μM for the WT) and a 4.7-fold increased affinity towards DAG-containing liposomes (*K*_*D*_ of 5.85 μM *versus* 27.8 μM for the WT) (Fig. S1). On the other hand, a triple mutant, bearing alanine substitutions in the highly conserved residues E86 and I89, together with the proximal S93, failed to bind 8:0 DAG and showed a 4.6-fold decreased affinity towards DAG-containing liposomes (*K*_*D*_ of 129 μM *versus* 27.8 μM for the WT) (Fig. 4*C*). To confirm the importance of the glutamic acid residue at position 86 for binding of DAG, we also generated an E86A point mutant version. This point mutant PLIN3 bound short chain 8:0 DAG with a *K*_*D*_ of 87.3 μM, that is 873-fold less efficient that did WT PLIN3 (*K*_*D*_ of 0.1 μM) (Fig. 4*C*). This point mutant PLIN3 also displayed a much higher dissociation constant for binding DAG-containing liposomes than did the WT protein (*K*_*D*_ of 243 μM of the E86A point mutant compared to a *K*_*D*_ of 27.8 μM for the WT) (Fig. 4*C*). These results thus corroborate the importance of these highly conserved residues within the third kink of the triangular-shaped PAT domain for binding of DAG and highlight a crucial function of E86 in DAG binding. It is interesting to note that the corresponding glutamic acid residue in PLIN2, E73, has previously been shown to reduce binding of PLIN2 to ALDs containing PI, possibly through unfavorable electrostatic interactions between this membrane proximal acidic residue and the negatively charged lipid (40).

### Conserved residues in the PAT domain of PLIN3 are important for binding to ALDs

**Figure 5:**
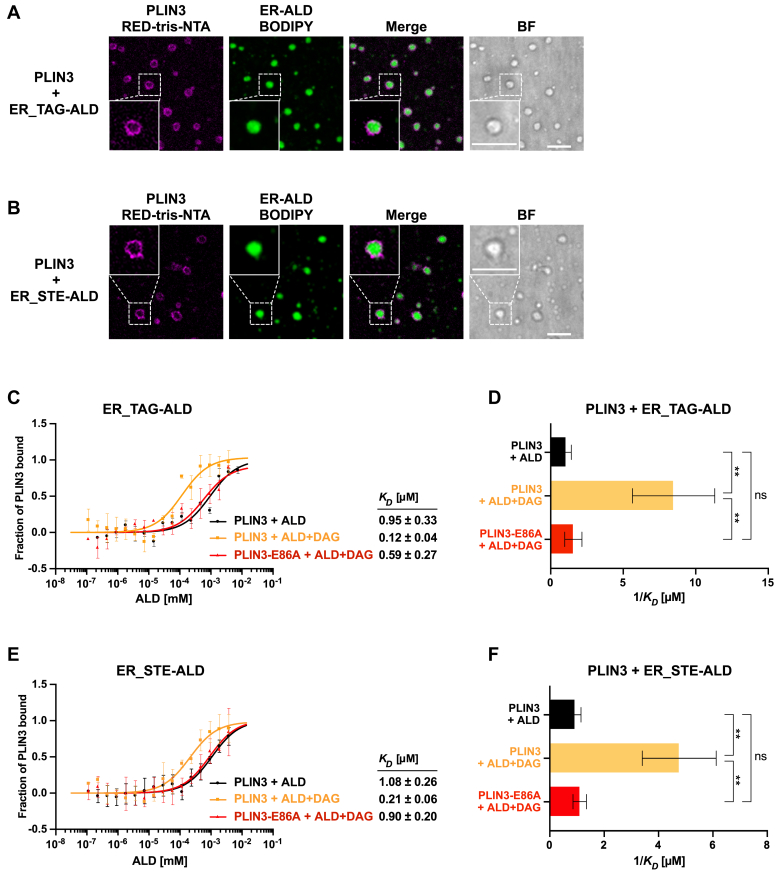
**Binding of PLIN3 to DAG-containing artificial lipid droplets is affected by the PAT domain.***A* and *B*, PLIN3 is recruited to the surface of the ALDs. Purified PLIN3 was fluorescently labeled with the RED-tris-NTA fluorophore and incubated with ALDs containing an ER-like phospholipid composition (DOPC/DOPE/DOPS/DOPA/SoyPI; 53/23/8/5/11 mol%) and either a hydrophobic core of TAG (triolein, ER_TAG-ALDs, *panel A*) or STE (cholesteryl oleate, ER_STE-ALDs, *panel B*). ALDs were stained with BODIPY, and fluorescence was imaged using a confocal microscope. The regions highlighted by the *white* boxes are shown at higher magnification in the panels below. Scale bar represents 5 μm. *C*–*F*, binding of WT and the E86A point mutant version of PLIN3 to TAG- or STE-containing ALDs with or without DAG. The fraction of bound protein is plotted against the ALD concentration and the dissociation constants (*K*_*D*_) are indicated (*panels C*, *E*). Values represent mean ± S.D. of three independent measurements. *K*_*D*_ determined in *C* and *E* are plotted as reciprocal values (*panels D*, *F*). One-way ANOVA was performed followed by Tukey's post hoc test (∗∗*p* < 0.01; ∗∗∗*p* < 0.001; ns, not significant). ALD, artificial LD; BF, brightfield visualization; DAG, diacylglycerol; ER, endoplasmic reticulum; NTA, nitrilotriacetic acid; PLIN, perilipin; STE, steryl ester; TAG, triacylglycerol.

The data so far indicate that the PAT domain of PLIN3 is important for targeting of the protein to bilayer membranes containing low concentrations of DAG. Given that PLIN3 is not only associating with ER membrane but specifically localizes to the surface monolayer of LDs, we examined binding of PLIN3 to ALDs, also known as adiposomes/nanodroplets (32, 40). These ALDs were generated using a lipid composition resembling that of the ER membrane to cover a hydrophobic core consisting either of TAG (triolein) or STE (cholesteryl oleate). When incubated together with ALDs, fluorescently labeled PLIN3 localized to the surface of both types of ALDs, as visualized by confocal microscopy (Fig. 5, *A* and *B*).

For a more quantitative assessment of the role of DAG in promoting binding of PLIN3 to the surface of ALDs and to assess the function of the PAT domain in ALD targeting, we used MST to measure the respective dissociation constants. PLIN3 bound to the surface of ALDs with nanomolar affinity irrespective of whether they contained a core of TAG or STE (*K*_*D*_ of 0.95 μM for ER_TAG-ALDs and 1.08 μM for ER_STE-ALDs; Fig. 5, *C*–*F*). These data indicate that PLIN3 binds to ALDs with >500-fold higher affinity than it binds bilayer membrane–containing liposomes (Figs. 1*C* and 5, *C* and *E*). The addition of low concentrations of DAG improved binding of PLIN3, irrespective of the nature of the hydrophobic core of the ALDs (*K*_*D*_ of 0.12 μM for ER_TAG-ALDs and 0.21 μM for ER_STE-ALDs; Fig. 5, *C*–*F*). The E86A point mutant version of PLIN3, however, displayed binding affinities to the DAG-containing ALDs that were similar to those of the WT protein towards ALDs lacking DAG (*K*_*D*_ of 0.59 μM for ER_TAG-ALDs and 0.90 μM for ER_STE-ALDs; Fig. 5, *C*–*F*). These data thus indicate that the E86A point mutation renders PLIN3 none-responsive towards DAG. The data are thus consistent with the notion that DAG recognition is mediated by the PAT domain of PLIN3 and that this domain can recognize DAG both when embedded in a bilayer as well as in a monolayer membrane.

### DAG impacts PLIN3 recruitment to LDs in yeast cells

**Figure 6:**
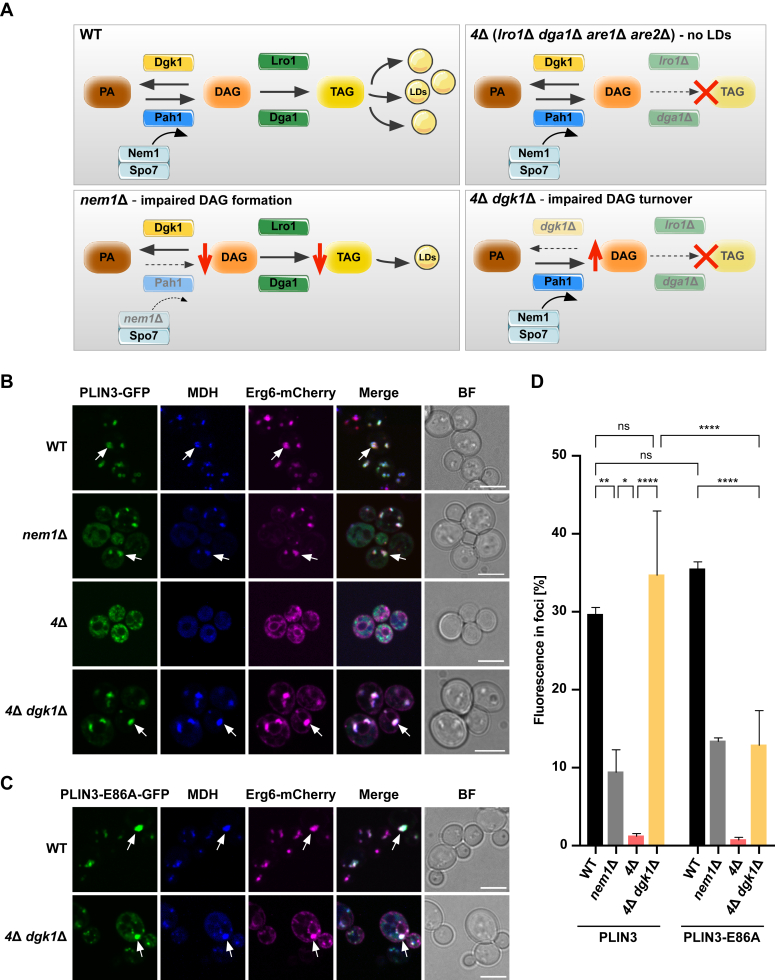
**DAG levels affect LD recruitment of PLIN3 in *S. cerevisiae*.***A*, schematic drawing of neutral lipid synthesis in WT cells and the impact of deletions of the indicated genes. *Red cross*, impaired TAG synthesis; *red downward arrow*, decreased levels of DAG/TAG; *red upward arrow*, increased levels of DAG. *B* and *C*, representative images of the indicated strains expressing GFP-tagged PLIN3 (*B*) or the PLIN3-E86A point mutant version (*C*), together with a genomic-tagged Erg6-mCherry. LDs were stained with the neutral lipid dye MDH. *Arrows* indicate the colocalization of the fluorescently labeled proteins with MDH-stained structures. Scale bars represent 5 μm. *D*, quantification of the fluorescence intensity of the GFP-tagged protein in foci plotted as the relative percentage of total cellular fluorescence. n > 80 cells/genotype. Data represent mean ± S.D. of three independent experiments. ∗*p* < 0.05; ∗∗*p* < 0.01; ∗∗∗∗*p* < 0.0001; ns, not significant (one-way ANOVA with Tukey's post hoc test). DAG, diacylglycerol; LD, lipid droplet; PA, phosphatidic acid; PLIN, perilipin; TAG, triacylglycerol.

To assess the importance of DAG for the recruitment of PLIN3 to LDs in cells, we analyzed the LD localization of GFP-tagged PLIN3 in a series of *Saccharomyces cerevisiae* strains bearing deletions of lipid biosynthetic genes (Fig. 6*A*). In a WT strain, PLIN3-GFP showed colocalization with LDs, as visualized by staining LDs with the neutral lipid dye MDH and the LD marker Erg6-mCherry (Fig. 6*B*), which is in line with previously described observations (9, 41). Quantitative analysis of PLIN3-GFP fluorescence signal indicates that 30% of total cellular fluorescence is concentrated in LD puncta in WT cells. In a *nem1Δ* mutant strain, however, LD-localized fluorescence of PLIN3-GFP is reduced by a factor of approximately 3 and cytosolic fluorescence is increased (Fig. 6, *B* and *D*). Nem1 is a component of the Nem1/Spo7 phosphatase complex, an activator of phosphatidate phosphatase Pah1, which converts PA into DAG. In the absence of Nem1, Pah1 is inactive, and this results in decreased levels of DAG (42, 43) (Fig. 6*A*). In cells that lack LDs, due to the deletion of the four neutral lipid biosynthetic genes (*4Δ*, *lro1Δ dga1Δ are1Δ are2Δ*), PLIN3-GFP was predominantly cytosolic, showing some staining of small granular structures, as described before (41) (Fig. 6, *B* and *D*). Deletion of the DAG kinase (Dgk1), which converts DAG into PA, in the 4Δ mutant background, however, resulted in an increased accumulation of PLIN3-GFP in LD-like punctate structures that were stained with both MDH and Erg6-mCherry (Fig. 6, *B* and *D*). This quintuple mutant is impaired in the conversion of DAG to either PA or to TAG, resulting in elevated levels of DAG (41, 44, 45). Thus, these LD-like structures appear to reassemble the DAG-containing membrane domains that we recently observed in cells expressing a membrane-anchored version of PLIN3 (46). Similar structures were also previously observed in yeast and mammalian cells supplemented with a membrane-permeable version of DAG (32, 41).

Given that DAG levels impact the recruitment of PLIN3-GFP to LDs, we next tested whether the E86A point mutant of PLIN3, which affects DAG binding and recruitment to DAG-containing liposomes and ALDs *in vitro*, would also affect its LD localization *in vivo*. In WT cells, PLIN3-E86A-GFP was still efficiently recruited to LDs (Fig. 6, *C* and *D*). In the quintuple mutant lacking both LDs and Dgk1, however, the recruitment of the mutant version of PLIN3 to the LD-like structures was significantly reduced (Fig. 6, *C* and *D*). Taken together, these observations are consistent with the results obtained by the *in vitro* assays and support the importance of DAG for the recruitment of PLIN3 to LDs and that of the glutamic acid residue at position 86 of the PAT domain of PLIN3 for the recognition of LD-like structures that are enriched in DAG.

## Discussion

**Figure 7:**
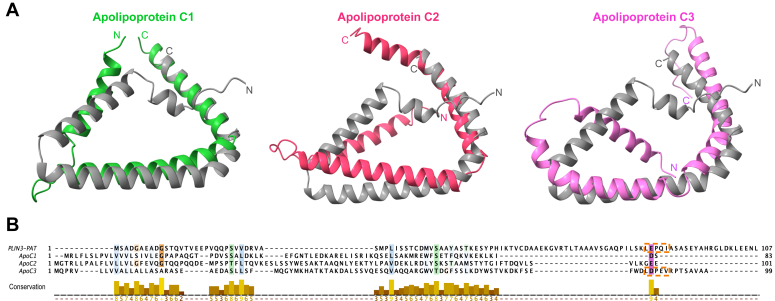
**The triangular shaped helical structure of the PAT domain is conserved in apolipoprotein C.***A*, superimposition of the triangular-shaped structure of the PAT domain of PLIN3 (*gray*) with those predicted for apolipoprotein C (ApoC). Proteins adopting a triangular structure similar to the PAT domain were identified by screening the distance matrix alignment (Dali) server (70). *B*, multiple sequence alignment of the PAT domain of PLIN3 with apolipoprotein C1, C2, and C3. The conserved LEPQI motif of the PAT domain and a highly similar LDPEV motif in ApoC-3 are indicated by *orange boxes*. PLIN, perilipin.

PLINs are soluble proteins that are targeted to the surface of LDs. How these proteins specifically recognize their target membrane is not fully understood (47). Here we show that PLIN3 exhibits increased binding towards membranes containing low concentrations of DAG. This specificity towards DAG-containing membranes is mediated by the N-terminal PAT domain of PLIN3, which is predicted to adopt a triangular-shaped structure composed of three AHs. This amphipathic triangular structure is likely conserved in other PAT-containing members of the PLIN family, particularly PLIN1, 2, and PLIN 5, but also in apolipoprotein C, the smallest exchangeable apolipoprotein of mammals (Fig. 7) (48). The PAT domain of PLIN3 which binds short chain 8:0 DAG *in vitro* and *in silico* docking experiments indicate that binding of DAG occurs at a conserved motif in a kinked region of the domain. Mutation of a conserved glutamic acid residue within this motif, E86, which is predicted to stabilize DAG through a hydrogen bridge, impairs lipid binding. Specificity of PLIN3 binding to DAG-containing membranes is not only observed with liposome bilayers but also with ALDs containing lipid monolayers. Binding of WT PLIN3 to the surface of ALDs occurs with an affinity that is three orders of magnitude higher than binding to bilayers and is further enhanced approximately 8-fold by the presence of DAG. Thus, a neutral lipid enclosing monolayer has by far the most profound effect on PLIN3 binding and this nanomolar binding affinity can be further enhanced by the presence of DAG.

The recognition of DAG by the PAT domain of PLIN3 is important for its enhanced binding affinity, as the E86A point mutant version of PLIN3 fails to show enhanced binding to DAG-containing ALDs. Thus, under these *in vitro* conditions, the recognition of DAG by the conserved motif within the PAT domain is important for enhancing binding of PLIN3 to DAG-containing ALDs. The other two domains present in PLIN3, the 11-mer repeat AHs and the 4-helix bundle, while they significantly contribute to the strong binding preference towards ALDs, they appear to be less important for the specificity towards DAG-containing ALDs (15, 49). This is consistent with the observation that the extremely long AH present in PLIN4, which lacks both the PAT domain and the 4-helix bundle, is sufficient for targeting of the protein to LDs in cells and to liposomes and ALDs *in vitro* (13). Such an overlapping binding specificity between the different domains of PLIN proteins may also explain some of the LD targeting specificities observed with truncated versions of PLINs *in vivo* (8, 9, 10, 18, 31, 49, 50). In addition, such an overlap in binding specificity between different domains has recently been experimentally supported by the observation that membrane binding by PLIN3 induces structural rearrangements within its PAT domain and the 11-mer repeats from disordered to ordered alpha helices (51).

The observed preference of PLIN3 to bind DAG is particularly interesting, as DAG has previously been shown to be required for LD formation, independently of its function as precursor to TAG synthesis (19, 20, 52). DAG is generally present at low concentrations in cellular membranes where it fulfills different functions. Yet, DAG concentrations in total cellular membranes vary greatly between different cell types and tissues (53). In yeast, DAG accounts for approximately 4 mol% of lipids and this concentration might increase locally either on formation of LDs in ER microdomains, upon lipolysis on LDs, or as a function of DAG signaling (20, 21, 54, 55). Indeed, DAG serves as the immediate precursor for the synthesis of the major storage lipid TAG and may accumulate at sites of TAG formation and LD biogenesis within the ER membrane, particularly at ER domains containing the LD biogenesis protein seipin (21, 23, 56). PLIN3 localizes to nascent LDs in the ER and the protein relocates to the ER upon addition of a membrane-permeable DAG (32, 41). On the other hand, DAG is also produced during lipolytic degradation of TAG, mediated by LD-localized lipases (55). Thus, a combination of protein domains harboring partially overlapping and potentially synergistic binding specificity for particular membrane/lipid features, such as DAG recognition by the PAT domain, the recognition of lipid packaging defects within a monolayer by the 11-mer repeats, and a possible binding preference for PE by the 4-helix bundle, may account for the observed strong specificity of PLIN3 to target LDs (31, 49).

While DAG in the ER would be embedded in a bilayer membrane, DAG produced through TAG lipolysis on LDs would localize to a phospholipid monolayer. Whether PLIN3 displays preference for bilayer or monolayer embedded DAG is an interesting question. Based on our *in vitro*–binding assays, the dissociation constant of PLIN3 for bilayer-embedded DAG is two orders of magnitudes higher than its binding to a DAG-containing monolayer (*K*_*D*_ of 20.6 μM for binding of PLIN3 to DAG-containing ER-liposomes *versus* a *K*_*D*_ of 0.12 μM for DAG-containing ER-ALDs; Figs. 1 and 5). Thus, based on these dissociation constants, one would expect a 170-fold preference of PLIN3 to localize to the surface of LDs compared to the ER membrane.

DAG is not only metabolically predestined to mark sites of LD biogenesis in the ER and the surface monolayer of LDs, but it has also unique biophysical properties. As an uncharged lipid with a small headgroup, DAG exhibits a conical shape. It promotes the formation of an inverted hexagonal phase and, at low concentrations, sinks into the hydrocarbon region of a phospholipid membrane, thereby inducing lipid packaging defects and exposing hydrocarbon chains of phospholipids (57, 58, 59). This balance between the propensity of DAG to induce phase transition and lipid packaging defects might also explain why PLIN3 displays optimal binding to liposomes containing 5 mol% of DAG but no further stimulation with higher DAG concentrations (Fig. 1*D*). NMR data indicate that DAG concentrations above 8 mol% within PC vesicles result in increased hydration of the lipid (60). These data suggest that PLIN3 preferentially recognizes nonhydrated DAG within a bilayer, which is consistent with the proposed hydrogen bridge between the primary alcohol of DAG and the glutamic acid residue at position 86 of PLIN3 (Fig. 4*B*). Whereas lipid packaging defects within a phospholipid monolayer are important for targeting the 11-mer repeat containing AH to the surface of nascent or mature LDs, recognition of DAG within a phospholipid bilayer by the PAT domain of PLIN3 further enhances binding affinity by 5 to 8-fold and might be important to recognize sites of LD biogenesis within the ER (21). The maximal binding affinity observed here with 5 mol% DAG are compatible with the physiological concentrations of DAG within membrane domains (53, 54, 55).

It is interesting to note that a similar high affinity binding to membranes containing low concentrations of DAG has previously been observed for the exchangeable apolipophorin III (61). Apolipophorins are lipoproteins that are made by insects to bind and transport lipids in their hemolymph. However, unlike PLINs or apolipoprotein C, the insect apolipophorins do not contain a PAT-like domain but instead possess a 4/5-helix domain containing AHs as present in ApoE and ApoAI, suggesting that this lipid-binding domain can also recognize DAG-containing membranes (17).

PLINs are both abundant and complex proteins that are likely important to fulfill a variety of functions, not only in regulating the access and activity of lipases but also by stabilizing the LD surface, preventing the coalescence of LDs, and promoting their formation from the ER membrane (32, 41, 62). Their typical three domain structure with an N-terminal PAT domain, a central 11-mer repeat AH, and a C-terminal 4-helix bundle has likely evolved to provide these functions under various physiological conditions. Defining the contribution of each one of these different protein domains and their potential synergistic interactions to the overall function of PLINs will likely provide important insights into the mode of action of this class of proteins.

## Experimental procedures

### Materials

Unless otherwise noted, lipids used in this study were purchased from Avanti Polar Lipids. The following short-chain lipids were used to test binding of more soluble lipids to PLIN3: 1,2-dioctanoyl-sn-glycero-3-phosphate sodium salt (8:0 PA, ≠830842), 1,2-diheptanoyl-sn-glycero-3-phosphocholine (7:0 PC, ≠850306), 1,2-dioctanoyl-sn-glycero-3-phospho-L-serine sodium salt (8:0 PS, ≠840031), 1,2-dioctanoyl-sn-glycerol (8:0 DAG, ≠800800), 1,2,3-trioctanoylglycerol (8:0 TAG, Merck, ≠T9126). For the preparation of liposomes, the following long-chain lipids were used: 1,2-dioleoyl-sn-glycero-3-phosphate (18:1 DOPA, ≠840875), 1,2-dioleoyl-sn-glycero-3-phosphocholine (18:1 DOPC, ≠850375), 1,2-dioleoyl-sn-glycero-3-phosphoethanolamine (18:1 DOPE, ≠850725), 1,2-dioleoyl-sn-glycero-3-phospho-L-serine sodium salt (18:1 DOPS, ≠840035), L-α-phosphatidylinositol sodium salt (Soy PI, ≠840044), 1,2-dipalmitoyl-sn-glycerol (16:0 DAG, ≠800816), 1,2-dioleoyl-sn-glycerol (18:1 DAG, ≠800811), cholesterol (Sigma; ≠C8667), 1,2-diphytanoyl-sn-glycero-3-phosphoethanolamine (4ME 16:0 PE, ≠850402), 1,2-diphytanoyl-sn-glycero-3-phosphocholine (4ME 16:0 PC, ≠850356), glyceryl trioleate (triolein, Merck, ≠T7140), cholesteryl oleate (Merck, ≠C9253), 1,2-dipentadecanoyl-rac-glycerol (15:0 DAG, Cayman Chemical, ≠26941).

### Yeast strains and culture conditions

The WT *S. cerevisiae* strain used in this study is BY4742 (*MATα*; *his3Δ1*; *leu2Δ0*; *lys2Δ0*; *ura3Δ0*, Euroscarf) containing a genomic-tagged version of *ERG6* (ERG6-mCherry). Mutants were derived from the same genetic background by deletion of the indicated genes using either PCR-based homologous recombination or by mating. Yeast cells were cultivated in YPD medium (1% bacto yeast extract, 2% bacto peptone, and 2% glucose) or selective media (0.67% yeast nitrogen base without amino acids, 2% glucose, and an amino acid drop-out mix) at 30 °C under agitation.

### Vector construction

Plasmids containing human full-length PLIN3 or fragments thereof were obtained by PCR amplification and all constructs were validated by sequencing (Microsynth AG). DNA amplification of PLIN3 fragments was carried out using KAPA HiFi DNA Polymerase (KAPA Biosystems, Roche). PCR products were cloned into *BamHI*/*NdeI*-digested pET16b vector (Novagen, Merck) using a Gibson Assembly Cloning Kit (New England Biolabs). Mutated versions of the PLIN3 gene were synthesized and cloned into pET16b by GenScript (Rijswijk). GFP-tagged PLIN3 was expressed from an *ADH1* promoter in the plasmid pGREG576 (pGREG576-ADH1-GFP-PLIN3) as was previously described (41). The E86A mutation was inserted in the pGREG576-ADH-GFP-PLIN3 plasmid.

### Protein expression and purification

Recombinant protein production and purification was performed using *Escherichia coli* BL21 (DE3) or NiCo21 (DE3) (New England Biolabs) strains. Transformed bacteria were grown in LB-ampicillin to an A_600_ of 0.5 at 37 °C. The expression of PLIN3 containing an N-terminal HIS-tag was induced with IPTG (0.75 mM), and cells were further cultivated at 30 °C for 2 h. Harvested cells were resuspended in lysis buffer composed of 50 mM Tris–HCl, pH 7.5, 300 mM NaCl, 20 mM imidazole, 10% glycerol, 1 mM PMSF, containing a complete EDTA-free protease inhibitor cocktail (PIC, Roche). Cells were disrupted using a Microfluidizer LM10 (Microfluidics), and the soluble fraction of the lysate was incubated with Ni^2+^-nitrilotriacetic acid (NTA) beads (Qiagen) at 4 °C for 2 h. Proteins were eluted with 300 mM imidazole in elution buffer (50 mM Tris–HCl, pH 7.5, 300 mM NaCl, 10% glycerol, 1 mM PMSF, and PIC). After elution, imidazole was removed by buffer exchange to 50 mM Tris–HCl, pH 7.5, 300 mM NaCl, 10% glycerol, 1 mM PMSF, and PIC, using Zeba spin desalting columns (Thermo Fisher Scientific). Protein concentration was determined by Lowry assay using Folin reagent and bovine serum albumin as standard.

### Liposome preparation

Lipids were dried from chloroform stock solutions using a rotary evaporator or under a gentle nitrogen gas stream. The dried lipid film was resuspended in 1 ml liposome buffer (50 mM NaCl, 25 mM Tris, pH 7.5) resulting in a concentration of 2 mM phospholipids. The phospholipid suspension was then subjected to ten cycles of freezing in liquid nitrogen and thawing in a water bath at 55 °C. The resulting multilamellar liposomes were extruded nineteen times through a polycarbonate filter of 0.8 μm pore size to generate large unilamellar vesicles. The size distribution of the large unilamellar vesicles was determined by dynamic light scattering (DLS, NanoLab 3D, LS Instruments AG). Liposomes showed a homogenous size distribution with a mean diameter between 90 and 200 nm. Dose-dependent incorporation of DAG into liposomes was verified by mass spectrometry (Q Exactive Orbitrap mass spectrometer, Thermo Fisher Scientific). DAG content was determined relative to that of an internal standard (15:0 DAG; Fig. S2).

### Preparation of ALDs

ALDs were generated as previously described (40). Two milligrams of total phospholipids in chloroform were dried under a gentle nitrogen gas stream. The phospholipids were hydrated with 100 μl of buffer B (20 mM Hepes, 100 mM KCl, 2 mM MgCl_2_, pH 7.4) and mixed with neutral lipid (triolein or cholesteryl oleate) in 1:2 M ratio. Cholesteryl oleate was heated to 50 °C to liquify the suspension (63). A crude lipid-buffer emulsion was generated by 24 cycles of vortexing, followed by 2 min of sonication. The lipid mixture was centrifuged at 1000*g* for 5 min at 4 °C, and large lipid particles floating on the surface as a white layer were removed. The solution underneath was collected and centrifuged again at 20,000*g* for 5 min at 4 °C to remove residual membrane debris. The size distribution of the ALDs was determined by dynamic light scattering. ALDs showed a homogenous size distribution with a mean diameter of 140 to 180 nm.

### Liposome flotation assay

Purified PLIN3 was incubated with liposomes for 1 h at RT and then gently mixed with an equal volume of 60% (w/v) sucrose solution in liposome buffer to obtain a final sucrose concentration of 30%. This mixture was overlaid with two volumes of 20% sucrose solution, two volumes of 10% sucrose solution, and one volume of liposome buffer. The samples were centrifuged at 177,000*g* for 1 h at 20 °C. Four fractions were collected from the top of the gradient and the distribution of PLIN3 was analyzed by Western blotting. Signal intensities were quantified using ImageJ (https://imagej.net/ij/) software.

### *In vitro* binding assays

Binding of purified PLIN3 protein and PLIN3 domains to liposomes, ALDs, or short chain lipids was determined by MST using a Monolith NT.115 system (Nanotemper Technologies). Proteins were labeled with a RED-tris-NTA His tag protein-labeling reagent following the manufacturer’s instructions (Nanotemper Technologies). Labeled proteins were added to a serial dilution of unlabeled lipids, liposomes, or ALDs, and the mixtures were loaded into MST standard capillaries to assess binding affinities. The dissociation constant was obtained by plotting fluorescence intensities of the protein–lipid complex against the concentrations of the unlabeled lipids, liposomes, or ALDs. Experiments were performed in triplicates and data were fitted using the *K*_*D*_ Model of the MO.Affinity Analysis software (https://nanotempertech.com/, Nanotemper Technologies).

### Fluorescent microscopy

To visualize the interaction of PLIN3 with ALDs, purified PLIN3 was labeled with the RED-tris-NTA His-tag labeling dye (Nanotemper Technologies). The protein was then incubated with ALDs for 1 h at RT and stained with BODIPY 493/503 (Invitrogen). ALDs were centrifuged at 20,000*g* for 5 min at 4 °C, the solution underneath the floating ALDs was removed, and ALDs were washed with 50 μl of Hepes buffer. This washing step was repeated three times, and a drop of the suspension was mounted on a glass slide for imaging.

Yeast cells expressing GFP-tagged PLIN3 were grown to early stationary phase and concentrated by centrifugation. LDs were stained with 50 μM MDH (≠SM1000a, Abcepta) for 15 min at 30 °C.

Images were acquired with a Visitron spinning disc CSU-W1 set-up (Visitron Systems), consisting of a Nikon Ti-E inverted microscope, equipped with a CSU-W1 spinning disk head (Yokogawa), an Evolve 512 (Photometrics) EM-CCD camera, and a PLAN APO 100× NA 1.3 oil objective (Nikon). Images were processed using ImageJ software.

For fluorescence quantification, all cells in an image were selected as regions of interest (cellular ROI) using the manual selection tool. The total fluorescence intensity within these cellular ROI was calculated as integrated intensity and corrected by subtracting the mean intensity of the background. In each cellular ROI, punctate GFP structures colocalizing with Erg6-mCherry or with MDH-labeled LDs were identified using Gaussian blur filtering and difference of Gaussians. This yielded a map of intracellular spots (punctuate ROI) with their integrated fluorescence intensity. The fluorescence intensity of punctuate ROI relative to that of cellular ROI corresponds to the percentage of total cellular PLIN3-GFP fluorescence localized to LDs. The data were statistically analyzed using GraphPad Prism 9.5.0 (https://www.graphpad.com/) software and plotted.

### *In silico* ligand docking

Ligand docking to PLIN3 was assessed using AutoDock Vina (38, 39). Since the tertiary structure of PLIN3 has not yet been experimentally determined, a structure predicted by AlphaFold2 was used (34, 35). The structures of lipids were obtained either from PubChem or ChemSpider (64) and converted to the PDB format using PyMOL (version 2.5.4, Schrödinger, LLC.). AutoDock Tools was then used to define the structures of PLIN3 as macromolecule target and that of the lipid as ligand for docking simulation and both files were converted to PDBQT format (65). Given the lack of information about possible lipid-binding sites on PLIN3, a blind docking was performed by generating an affinity map covering the entire protein. Each affinity map was then described by grid coordinates using AutoGrid 4. PLIN3 and a lipid in PDBQT format together with the corresponding grid coordinates were used as input for docking simulation by AutoDock Vina. The predicted positions of docked ligands and the corresponding bonding interactions were visualized using UCSF Chimera X (66).

## Data availability

All data is contained within the manuscript; then the statement should indicate so.

## Supporting information

This article contains supporting information.

## Conflict of interest

The authors declare that they have no conflicts of interest with the contents of this article.
