# Supplementary material for: Binding of perilipin 3 to membranes containing diacylglycerol is mediated by conserved residues within its PAT domain
Source: J Biol Chem. 2023 Oct 28;299(12):105384. doi: 10.1016/j.jbc.2023.105384 (PMC10694602; doi:10.1016/j.jbc.2023.105384)
Supplement: Supporting Figures S1 and S2 and Tables S1 and S2 [file mmc1.pdf]

Supporting Information

Supporting Figures

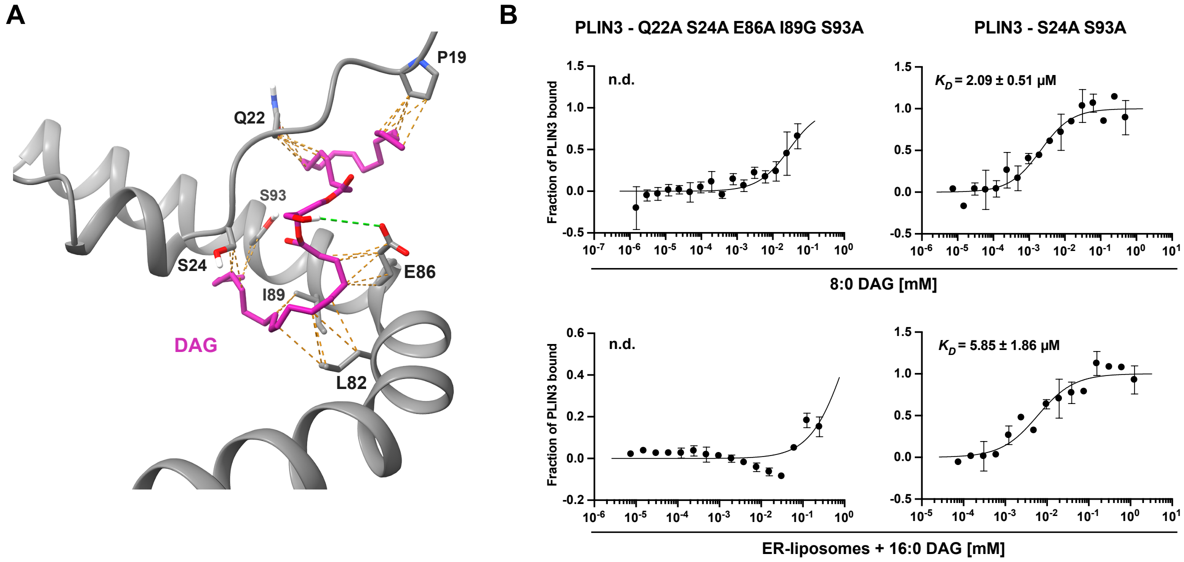

Figure S1. Impact of highly conserved residues within the PAT domain of PLIN3 on DAG binding.

A) Detailed view of the predicted interactions between DAG (pink) and residues within the PAT domain of PLIN3 (van der Waals interactions, dashed orange lines; hydrogen bonding, dashed green line). B) The impact of the indicated quintuple and double mutations on the binding of short chain DAG (8:0 DAG; *upper panels*) or ER-liposomes containing 16:0 DAG (5 mol%; *lower panels*) by PLIN3 was assessed by MST. Dissociation constants (K_D) represent mean ± S.D. of 3 independent measurements.

A

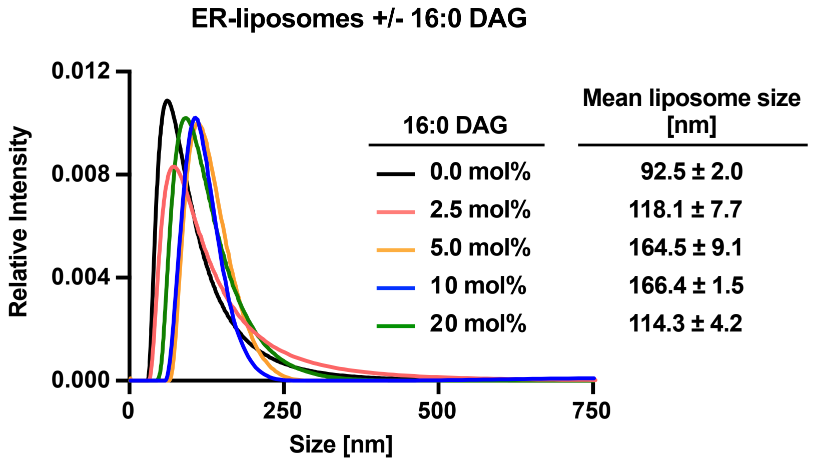

B

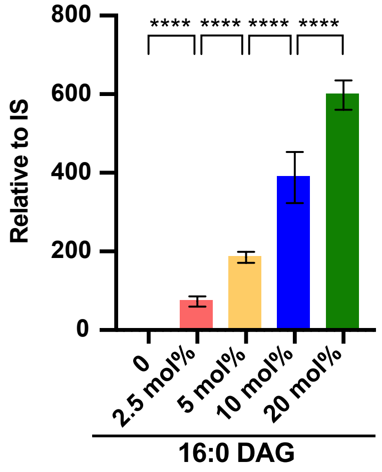

Figure S2. Analysis of liposomes containing increasing concentrations of DAG

A) DLS analysis of the size distribution of ER-liposomes composed of DOPC (53 mol%), DOPE (23 mol%), DOPS (8 mol%), DOPA (5 mol%) and SoyPI (11 mol%) lacking or containing 16:0 DAG (2.5 – 20 mol%). Values represent mean ± S.D. of three independent preparations. B) DAG levels in ER-liposomes were verified by mass spectrometry. Liposomes containing the indicated concentration of DAG were purified by flotation, spiked with an internal standard (IS; 15:0 DAG), lipids were extracted and C16:0 DAG levels were measured by mass spectrometry. Data represent mean ± S.D. of three independent measurements. ****, $p < 0.0001$ (one-way ANOVA with Tukey's post hoc test).

Supporting Tables

Table S1. Precise *p*-values for the statistical comparisons shown in figures 1, 5, and 6.
Data indicated in the corresponding figures are highlighted in bold.

Figure 1B. PLIN3 distribution in sucrose fractions

Two tailed unpaired t-test	Adjusted P Value
**No liposomes vs. ER**	**0.0121**
**ER vs. ER + 16:0 DAG**	**0.0046**
No liposomes vs. ER + 16:0 DAG	0.0013

Figure 1D. PLIN3 + ER-liposomes ± 16:0 DAG

Tukey's multiple comparisons test	Adjusted P Value
0 vs. 2.5 mol%	0.1656
**0 vs. 5 mol%**	**0.0002**
0 vs. 10 mol%	0.0288
0 vs. 20 mol%	0.1442
**2.5 mol% vs. 5 mol%**	**0.0049**
2.5 mol% vs. 10 mol%	0.7818
2.5 mol% vs. 20 mol%	>0.9999
**5 mol% vs. 10 mol%**	**0.0275**
**5 mol% vs. 20 mol%**	**0.0056**
10 mol% vs. 20 mol%	0.8277

Figure 1F. PLIN3 + ER-liposomes ± 16:0 DAG

Tukey's multiple comparisons test	Adjusted P Value
**0 vs. 5 mol%**	**0.9323**
**0 vs. 10 mol%**	**0.9860**
**0 vs. 20 mol%**	**0.0115**
0 vs. 5 mol% + 5 mol% DAG	0.0213
**5 mol% vs. 10 mol%**	**0.9984**
**5 mol% vs. 20 mol%**	**0.0378**
5 mol% vs. 5 mol% + 5 mol% DAG	0.0706
**10 mol% vs. 20 mol%**	**0.0246**
10 mol% vs. 5 mol% + 5 mol% DAG	0.0459
**20 mol% vs. 5 mol% + 5 mol% DAG**	**0.9935**

Figure 1G. PLIN3 + ER-liposomes ± DiphPC/PE

Tukey's multiple comparisons test	Adjusted P Value
no DAG 0 vs. no DAG 5	0.9972
no DAG 0 vs. no DAG 10	0.0355
no DAG 0 vs. no DAG 20	0.0007
**no DAG 0 vs. 16:0 DAG 0**	**0.0048**
no DAG 0 vs. 16:0 DAG 5	<0.0001
no DAG 0 vs. 16:0 DAG 10	0.0028
no DAG 0 vs. 16:0 DAG 20	0.0048
no DAG 5 vs. no DAG 10	0.0466
no DAG 5 vs. no DAG 20	0.0067
no DAG 5 vs. 16:0 DAG 0	>0.9999
**no DAG 5 vs. 16:0 DAG 5**	**<0.0001**
no DAG 5 vs. 16:0 DAG 10	0.0066
no DAG 5 vs. 16:0 DAG 20	>0.9999
no DAG 10 vs. no DAG 20	0.0511
no DAG 10 vs. 16:0 DAG 0	0.9929
no DAG 10 vs. 16:0 DAG 5	<0.0001
**no DAG 10 vs. 16:0 DAG 10**	**0.5979**
no DAG 10 vs. 16:0 DAG 20	0.9944
no DAG 20 vs. 16:0 DAG 0	0.6376
no DAG 20 vs. 16:0 DAG 5	<0.0001
no DAG 20 vs. 16:0 DAG 10	0.9885
**no DAG 20 vs. 16:0 DAG 20**	**0.0087**
**16:0 DAG 0 vs. 16:0 DAG 5**	**<0.0001**
16:0 DAG 0 vs. 16:0 DAG 10	0.0053
16:0 DAG 0 vs. 16:0 DAG 20	0.9981
**16:0 DAG 5 vs. 16:0 DAG 10**	**0.0001**
**16:0 DAG 5 vs. 16:0 DAG 20**	**<0.0001**
16:0 DAG 10 vs. 16:0 DAG 20	0.0056

Figure 1H. PLIN3 + PC/PE-liposomes

Tukey's multiple comparisons test	Adjusted P Value
**DOPC-DOPE vs. 16:0 DAG**	**0.0097**
**DOPC-DOPE vs. DOPA**	**0.5657**
**16:0 DAG vs. DOPA**	**0.0321**

Figure 1I. PLIN3 + Short chain lipids

Tukey's multiple comparisons test	Adjusted P Value
**7:0 PC vs. 8:0 DAG**	**<0.0001**
7:0 PC vs. 8:0 PS	>0.9999
**7:0 PC vs. 8:0 PA**	**>0.9999**
**7:0 PC vs. 8:0 TAG**	**0.0060**
8:0 DAG vs. 8:0 PS	<0.0001
**8:0 DAG vs. 8:0 PA**	**<0.0001**
**8:0 DAG vs. 8:0 TAG**	**<0.0001**
8:0 PS vs. 8:0 PA	>0.9999
8:0 PS vs. 8:0 TAG	0.0061
8:0 PA vs. 8:0 TAG	0.0062

Figure 5D. PLIN3 + ER_TAG-ALD

Tukey's multiple comparisons test	Adjusted P Value
**PLIN3+ALD vs. PLIN3+ALD+DAG**	**0.0044**
**PLIN3+ALD vs. PLIN3-E86A+ALD+DAG**	**0.8939**
**PLIN3+ALD+DAG vs. PLIN3-E86A+ALD+DAG**	**0.0068**

Figure 5F. PLIN3 + ER_STE-ALD

Tukey's multiple comparisons test	Adjusted P Value
**PLIN3+ALD vs. PLIN3+ALD+DAG**	**0.0028**
**PLIN3+ALD vs. PLIN3-E86A+ALD+DAG**	**0.9579**
**PLIN3+ALD+DAG vs. PLIN3-E86A+ALD+DAG**	**0.0036**

Figure 6D. Quantification of the fluorescence intensity

Tukey's multiple comparisons test	Adjusted P Value
**WT vs. nem1Δ**	**<0.0001**
**WT vs. 4Δ dgk1Δ**	**0.6487**
WT vs. 4Δ	<0.0001
WT vs. E86A 4Δ dgk1Δ	<0.0001
**WT vs. E86A WT**	**0.4886**
WT vs. E86A nem1Δ	0.0004
WT vs. E86A 4Δ	<0.0001
nem1Δ vs. 4Δ dgk1Δ	<0.0001
**nem1Δ vs. 4Δ**	**0.0367**
nem1Δ vs. E86A 4Δ dgk1Δ	0.8719
nem1Δ vs. E86A WT	<0.0001
nem1Δ vs. E86A nem1Δ	0.8581
nem1Δ vs. E86A 4Δ	0.0672
**4Δ dgk1Δ vs. 4Δ**	**<0.0001**
**4Δ dgk1Δ vs. E86A 4Δ dgk1Δ**	**<0.0001**
4Δ dgk1Δ vs. E86A WT	>0.9999
4Δ dgk1Δ vs. E86A nem1Δ	<0.0001
4Δ dgk1Δ vs. E86A 4Δ	<0.0001
4Δ vs. E86A 4Δ dgk1Δ	0.0044
4Δ vs. E86A WT	<0.0001
4Δ vs. E86A nem1Δ	0.0086
4Δ vs. E86A 4Δ	>0.9999
**E86A 4Δ dgk1Δ vs. E86A WT**	**<0.0001**
E86A 4Δ dgk1Δ vs. E86A nem1Δ	>0.9999
E86A 4Δ dgk1Δ vs. E86A 4Δ	0.0012
E86A WT vs. E86A nem1Δ	<0.0001
E86A WT vs. E86A 4Δ	<0.0001
E86A nem1Δ vs. E86A 4Δ	0.0031

Table S2. Precise *p*-values for the statistical comparisons shown in figure 2.
Data indicated in the corresponding figures are highlighted in bold.

Figure 2B. PLIN3 domains + Short chain lipids

Tukey's multiple comparisons test	Adjusted P Value
4helix:7:0 PC vs. 4helix:8:0 PS	>0.9999
4helix:7:0 PC vs. 4helix:8:0 PA	>0.9999
4helix:7:0 PC vs. 4helix:8:0 TAG	>0.9999
**4helix:7:0 PC vs. 4helix:8:0 DAG**	**>0.9999**
4helix:7:0 PC vs. 11mer:7:0 PC	>0.9999
4helix:7:0 PC vs. 11mer:8:0 PS	>0.9999
4helix:7:0 PC vs. 11mer:8:0 PA	>0.9999
4helix:7:0 PC vs. 11mer:8:0 TAG	>0.9999
4helix:7:0 PC vs. 11mer:8:0 DAG	>0.9999
4helix:7:0 PC vs. PAT:7:0 PC	>0.9999
4helix:7:0 PC vs. PAT:8:0 PS	>0.9999
4helix:7:0 PC vs. PAT:8:0 PA	>0.9999
4helix:7:0 PC vs. PAT:8:0 TAG	>0.9999
4helix:7:0 PC vs. PAT:8:0 DAG	<0.0001
4helix:8:0 PS vs. 4helix:8:0 PA	>0.9999
4helix:8:0 PS vs. 4helix:8:0 TAG	>0.9999
4helix:8:0 PS vs. 4helix:8:0 DAG	>0.9999
4helix:8:0 PS vs. 11mer:7:0 PC	>0.9999
4helix:8:0 PS vs. 11mer:8:0 PS	>0.9999
4helix:8:0 PS vs. 11mer:8:0 PA	>0.9999
4helix:8:0 PS vs. 11mer:8:0 TAG	>0.9999
4helix:8:0 PS vs. 11mer:8:0 DAG	>0.9999
4helix:8:0 PS vs. PAT:7:0 PC	>0.9999
4helix:8:0 PS vs. PAT:8:0 PS	>0.9999
4helix:8:0 PS vs. PAT:8:0 PA	>0.9999
4helix:8:0 PS vs. PAT:8:0 TAG	>0.9999
4helix:8:0 PS vs. PAT:8:0 DAG	<0.0001
4helix:8:0 PA vs. 4helix:8:0 TAG	>0.9999
4helix:8:0 PA vs. 4helix:8:0 DAG	>0.9999
4helix:8:0 PA vs. 11mer:7:0 PC	>0.9999
4helix:8:0 PA vs. 11mer:8:0 PS	>0.9999
4helix:8:0 PA vs. 11mer:8:0 PA	>0.9999
4helix:8:0 PA vs. 11mer:8:0 TAG	>0.9999
4helix:8:0 PA vs. 11mer:8:0 DAG	>0.9999
4helix:8:0 PA vs. PAT:7:0 PC	>0.9999
4helix:8:0 PA vs. PAT:8:0 PS	>0.9999
4helix:8:0 PA vs. PAT:8:0 PA	>0.9999
4helix:8:0 PA vs. PAT:8:0 TAG	>0.9999
4helix:8:0 PA vs. PAT:8:0 DAG	<0.0001
4helix:8:0 TAG vs. 4helix:8:0 DAG	>0.9999
4helix:8:0 TAG vs. 11mer:7:0 PC	>0.9999
4helix:8:0 TAG vs. 11mer:8:0 PS	>0.9999
4helix:8:0 TAG vs. 11mer:8:0 PA	>0.9999
4helix:8:0 TAG vs. 11mer:8:0 TAG	>0.9999
4helix:8:0 TAG vs. 11mer:8:0 DAG	>0.9999
4helix:8:0 TAG vs. PAT:7:0 PC	>0.9999
4helix:8:0 TAG vs. PAT:8:0 PS	>0.9999
4helix:8:0 TAG vs. PAT:8:0 PA	>0.9999
4helix:8:0 TAG vs. PAT:8:0 TAG	>0.9999
4helix:8:0 TAG vs. PAT:8:0 DAG	<0.0001
4helix:8:0 DAG vs. 11mer:7:0 PC	>0.9999
4helix:8:0 DAG vs. 11mer:8:0 PS	>0.9999
4helix:8:0 DAG vs. 11mer:8:0 PA	>0.9999
4helix:8:0 DAG vs. 11mer:8:0 TAG	>0.9999
4helix:8:0 DAG vs. 11mer:8:0 DAG	>0.9999
4helix:8:0 DAG vs. PAT:7:0 PC	>0.9999
4helix:8:0 DAG vs. PAT:8:0 PS	>0.9999
4helix:8:0 DAG vs. PAT:8:0 PA	>0.9999
4helix:8:0 DAG vs. PAT:8:0 TAG	>0.9999
**4helix:8:0 DAG vs. PAT:8:0 DAG**	**<0.0001**

Figure 2B. PLIN3 domains + Short chain lipids

Tukey's multiple comparisons test	Adjusted P Value
11mer:7:0 PC vs. 11mer:8:0 PS	>0.9999
11mer:7:0 PC vs. 11mer:8:0 PA	>0.9999
11mer:7:0 PC vs. 11mer:8:0 TAG	>0.9999
11mer:7:0 PC vs. 11mer:8:0 DAG	>0.9999
11mer:7:0 PC vs. PAT:7:0 PC	>0.9999
11mer:7:0 PC vs. PAT:8:0 PS	>0.9999
11mer:7:0 PC vs. PAT:8:0 PA	>0.9999
11mer:7:0 PC vs. PAT:8:0 TAG	>0.9999
11mer:7:0 PC vs. PAT:8:0 DAG	<0.0001
11mer:8:0 PS vs. 11mer:8:0 PA	>0.9999
11mer:8:0 PS vs. 11mer:8:0 TAG	>0.9999
11mer:8:0 PS vs. 11mer:8:0 DAG	>0.9999
11mer:8:0 PS vs. PAT:7:0 PC	>0.9999
11mer:8:0 PS vs. PAT:8:0 PS	>0.9999
11mer:8:0 PS vs. PAT:8:0 PA	>0.9999
11mer:8:0 PS vs. PAT:8:0 TAG	>0.9999
11mer:8:0 PS vs. PAT:8:0 DAG	<0.0001
11mer:8:0 PA vs. 11mer:8:0 TAG	>0.9999
11mer:8:0 PA vs. 11mer:8:0 DAG	>0.9999
11mer:8:0 PA vs. PAT:7:0 PC	>0.9999
11mer:8:0 PA vs. PAT:8:0 PS	>0.9999
11mer:8:0 PA vs. PAT:8:0 PA	>0.9999
11mer:8:0 PA vs. PAT:8:0 TAG	>0.9999
11mer:8:0 PA vs. PAT:8:0 DAG	<0.0001
11mer:8:0 TAG vs. 11mer:8:0 DAG	>0.9999
11mer:8:0 TAG vs. PAT:7:0 PC	>0.9999
11mer:8:0 TAG vs. PAT:8:0 PS	>0.9999
11mer:8:0 TAG vs. PAT:8:0 PA	>0.9999
11mer:8:0 TAG vs. PAT:8:0 TAG	>0.9999
11mer:8:0 TAG vs. PAT:8:0 DAG	<0.0001
11mer:8:0 DAG vs. PAT:7:0 PC	>0.9999
11mer:8:0 DAG vs. PAT:8:0 PS	>0.9999
11mer:8:0 DAG vs. PAT:8:0 PA	>0.9999
11mer:8:0 DAG vs. PAT:8:0 TAG	>0.9999
11mer:8:0 DAG vs. PAT:8:0 DAG	<0.0001
PAT:7:0 PC vs. PAT:8:0 PS	>0.9999
PAT:7:0 PC vs. PAT:8:0 PA	>0.9999
PAT:7:0 PC vs. PAT:8:0 TAG	>0.9999
PAT:7:0 PC vs. PAT:8:0 DAG	<0.0001
PAT:8:0 PS vs. PAT:8:0 PA	>0.9999
PAT:8:0 PS vs. PAT:8:0 TAG	>0.9999
PAT:8:0 PS vs. PAT:8:0 DAG	<0.0001
PAT:8:0 PA vs. PAT:8:0 TAG	>0.9999
**PAT:8:0 PA vs. PAT:8:0 DAG**	**<0.0001**
**PAT:8:0 TAG vs. PAT:8:0 DAG**	**<0.0001**

Figure 2C. PLIN3 domains + ER-liposomes

Tukey's multiple comparisons test	Adjusted P Value
4helix:ER - liposomes vs. 4helix:ER-16:0 DAG	0.5951
4helix:ER - liposomes vs. 11mer:ER - liposomes	0.9133
4helix:ER - liposomes vs. 11mer:ER-16:0 DAG	0.3614
4helix:ER - liposomes vs. PAT:ER - liposomes	>0.9999
4helix:ER - liposomes vs. PAT:ER-16:0 DAG	<0.0001
4helix:ER-16:0 DAG vs. 11mer:ER - liposomes	0.9849
**4helix:ER-16:0 DAG vs. 11mer:ER-16:0 DAG**	**0.9974**
4helix:ER-16:0 DAG vs. PAT:ER - liposomes	0.5951
**4helix:ER-16:0 DAG vs. PAT:ER -16:0 DAG**	**<0.0001**
**11mer:ER - liposomes vs. 11mer:ER-16:0 DAG**	**0.8788**
11mer:ER - liposomes vs. PAT:ER - liposomes	0.9133
11mer:ER - liposomes vs. PAT:ER-16:0 DAG	<0.0001
11mer:ER-16:0 DAG vs. PAT:ER - liposomes	0.3614
**11mer:ER-16:0 DAG vs. PAT:ER -16:0 DAG**	**<0.0001**
PAT:ER - liposomes vs. PAT:ER-16:0 DAG	<0.0001
